# Early prey intake of a short‐finned pilot whale (*Globicephala macrorhynchus* Gray, 1846, Cetacea: Delphinidae) in the Canary Islands

**DOI:** 10.1002/ece3.11139

**Published:** 2024-03-10

**Authors:** Amanda Luna, Alejandro Escánez, Jacobo Marrero, Eva Íñiguez, José A. Pérez, Pilar Sánchez

**Affiliations:** ^1^ BioCephaLab Centro de Investigación Mariña de la Universidade de Vigo, Edificio de Ciencias Experimentais Vigo Spain; ^2^ Departamento de Ecoloxía e Bioloxía Animal, Edificio de Ciencias Experimentais, Campus As Lagoas‐Marcosende Universidade de Vigo Vigo Spain; ^3^ MARE‐Marine and Environmental Sciences Centre ARDITI, Edifício Madeira Tecnopolo, Caminho da Penteada Funchal Madeira Island Portugal; ^4^ Asociación Tonina San Cristóbal de La Laguna Tenerife (Islas Canarias) Spain; ^5^ Faculty of Life Sciences University of Madeira Funchal Madeira Island Portugal; ^6^ Departamento de Biología Animal, Edafología y Geología Universidad de La Laguna San Cristóbal de La Laguna Spain; ^7^ Department of Marine Renewable Resources Institute of Marine Sciences‐CSIC Barcelona Spain

**Keywords:** fatty acids, *Globicephala macrorhynchus*, Macaronesia, squids, stable isotopes, stomach contents, teuthophagous diet

## Abstract

This study reveals early prey eating by a short‐finned pilot whale (*Globicephala macrorhynchus* Gray, 1846, Cetacea: Delphinidae) in the Canary Islands. Stomach contents, trophic markers, skin isotopic ratios of nitrogen (δ^15^N:^15^N/^14^N) and carbon (δ^13^C:^13^C/^12^C), and fatty acid profiles of the blubber of a short‐finned pilot whale of 213 cm size euthanized in free‐ranging conditions were analyzed. A total of 15 species of oegopsid squids, mostly diel vertical mesopelagic migrant species of the families Enoploteuthidae, Ommastrephidae, and Histioteuthidae, as well as mother's milk, were identified in the stomach contents. *Asperoteuthis acanthoderma* (Lu, 1977, Cephalopoda: Chiroteuthidae) was found as first time in this area, suggesting the possibility of its presence on both sides of the subtropical Atlantic, extending its current known distribution. The δ^15^N value (11.55‰) was higher than expected based on the size range of squid ingested, but lower than that of adult pilot whales, suggesting that mother's milk intake has a significant effect on these values in calves. Similarly, the δ^13^C values (−17.99‰) were shifted to those of adult pilot whales rather than the ingested squids, also due to the ingestion of high‐fat breast milk. The fatty acid (FA) composition of blubber showed a clear stratification. Long‐chain polyunsaturated fatty acids (LC‐PUFA) were mainly present in the inner layer, while most relevant ≤C20 monounsaturated fatty acids (MUFA) were more abundant in the outer layer.

## Graphical abstract

The short‐finned pilot whale, *Globicephala macrorhynchus* in the Canary Islands. Stomach contents, trophic markers, skin isotopic ratios of nitrogen (δ^15^N: ^15^N/^14^N) and carbon (δ^13^C: ^13^C/^12^C), and fatty acid profiles of the blubber of a short‐finned pilot whale were analyzed to obtain the insights in their early hunting behavior.

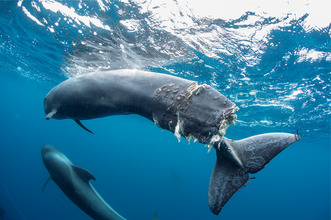

## 1 INTRODUCTION

The ontogeny of hunting behavior in cetaceans, which involves the development of cognitive, echolocation and diving skills, remains poorly understood for most cetaceans, especially for deep diving odontocetes. First, prey's intake in calves of deep diving odontocetes remains widely unknown and, to the best of our knowledge, this sort of data is restricted to some of them: the sperm whale (*Physeter macrocephalus* Linnaeus, 1758, Cetacea: Physeteridae), the Risso's Dolphin (*Grampus griseus* G. Cuvier, 1812, Cetacea: Delphinidae), the Baird beaked whale (*Berardius bairdii* Stejneger, 1883, Cetacea: Ziphiidae), and the southern bottlenose whale (*Hyperoodon planifrons* Flower, 1882, Cetacea: Ziphiidae). They are all teuthofagous species, i.e. they feed mainly on oceanic cephalopod species (Dixon et al., 1994; Luna et al., 2022; Santos et al., 2006; Walker et al., 2002). It has been documented that the calves of some marine mammals complement their lactation with solid food during their early life cycle, as occur in Risso's dolphins, which feed on oceanic cephalopods while continuing suckling (Blanco et al., 2006). However, detailed data on cephalopods consumed by these young specimens of deep diving cetaceans are not treated individually in the published articles so hampering the understanding of their early trophic ecology. The first solid feeding in these species seems related with the development of the diving capacities as well as their echolocation skills. In this sense, sperm whales have demonstrated early aptness to perform deep foraging dives below 600 m depth (Tønnesen et al., 2018). In addition, Best et al. (1984) found solid food items in the stomach contents of a one‐year‐old sperm whale, which may reflect independent foraging or food provisioning by adults. Biologging studies have revealed that other deep divers such as Blainville's beaked whale (*Mesoplodon densirostris* de Blainville, 1817, Cetacea: Ziphiidae) show highly synchronization in adult diving behavior, which is consistent with observations made from surface, where all group members dive and re‐surface in close coordination (Aguilar de Soto et al., 2020). Young members of these groups, estimated around 3 months, 18 months, and 2 years old, produce clicks in the same way adults and appear to always remain with their mothers, diving and surfacing in synchrony (Dunn et al., 2017), which could indicate a similar capacity for diving and foraging as the adults.

Among deep diving odontocetes, the short‐finned pilot whale (*Globicephala macrorhynchus* Gray, 1846, Cetacea: Delphinidae), herein after referred to as pilot whale, has been described as a mainly teutophagous species that can supplement its diet with fish and crustaceans (Öztürk et al., 2007; Riccialdelli et al., 2013). Thus, its distribution and habitat use have been related to the distribution, abundance and ease of capture of its main food resource, squids (e.g., Copeland et al., 2019; Hui, 1985; Owen et al., 2019; Seagars & Henderson, 1985; Sinclair, 1992). However, the pilot whales have shown a plasticity to adapt their feeding behavior to regional conditions (Shearer et al., 2022). This plasticity explains why they feed in separate regions on ecologically different squid species (from neritic to mesopelagic), and why they can prey on fish in considerable quantities in some areas (Bustamante et al., 2003; Mintzer et al., 2008). Despite this available information, few stomach contents of this species have been analyzed and published worldwide (Fernández et al., 2009; Mintzer et al., 2008; Overholtz & Waring, 1991; Seagars & Henderson, 1985), which means that there is still limited knowledge of its diet in the different regions where it is found, and even more with regard to the youngest individuals. There are also scarce studies on the diet or trophic ecology of this species using trophic biomarkers such as stable isotopes and fatty acid (FA) profiling, which have been widely used for other cetaceans (e.g., Bowen & Iverson, 2013). Stable isotopes, particularly of δ^13^C and δ^15^N, are commonly used in ecological studies, the first to determine the carbon source at the base of the food chain and the feeding habitat of individuals, and latter to study the trophic position of the individuals in relation to their prey (Xavier et al., 2022). This technique, when used on cephalopod beaks, has some advantages, such as that the stable isotopic composition is independent of the period of time during which the beak has been preserved in collections, and that the method of preservation (e.g. dried, frozen, in ethanol, in formalin) does not affect the results, being comparable for different types of studies (Xavier et al., 2022). Fatty acid composition is used to analyze some important dietary parameters of the species, such as final biomass, specific growth rate, or feed intake (Torres et al., 2013) according to the consumption of different kind of food, by being able to compare the nutritional nature of the ecological niche of preys.

In this sense, trophic ecology based on fresh tissues isotopic values of pilot whales have only been reported for few regions, including Gulf of California (Western Pacific), Moorea Island (South Pacific), and the Iberian Peninsula (Northwest Atlantic) (Aurioles‐Gamboa et al., 2013; Kiszka et al., 2010; Monteiro et al., 2017).

Tenerife island (Spain) holds one of the few resident populations of pilot whales worldwide, jointly with other oceanic islands such as Madeira and Hawaii (Alves et al., 2013; Heimlich‐Boran, 1993; Servidio et al., 2019; Shane & McSweeney, 1990). Previous works on adult pilot whales from Tenerife using motion and digital acoustic recording tags (DTAGs) (Johnson & Tyack, 2003), have shown that these animals feed at average depths between 810 ± 92 m during the day, and 141 ± 81 m at night (Aguilar de Soto et al., 2008). It has been suggested a daytime foraging tactic focused on large, calorific, and mobile prey items, such as large squids, that forces pilot whales to perform sprints up to 9 m/s in depths between 538 and 1019 m. In contrast, during night time, foraging dives are shallower and are characterized by steady swimming speeds and multiple prey capture attempts per dive (Aguilar de Soto et al., 2008). However, little is known about the stomach contents of this species in the Canary Islands, being impossible to confirm this hypothesis and, therefore, to have a better understanding of the population feeding habits. To date, only three stomach contents have been analyzed, showing remains of mesopelagic squids, mainly species of the Cranchiidae Prosch, 1847 family (Fernández et al., 2009; Hernández‐García & Martín, 1994).

Here, we present data on the simultaneous analyses of the stomach contents, bubbler stable isotopes, and FA signatures of a still lactating pilot whale calf found in the open sea off Tenerife Island. This case represents a unique opportunity to boost the understanding of the early hunting behavior and prey's selection of the offspring of this pilot whale population.

## 2 MATERIALS AND METHODS

### 2.1 Short‐finned pilot whale capture and euthanasia

**FIGURE 1:**
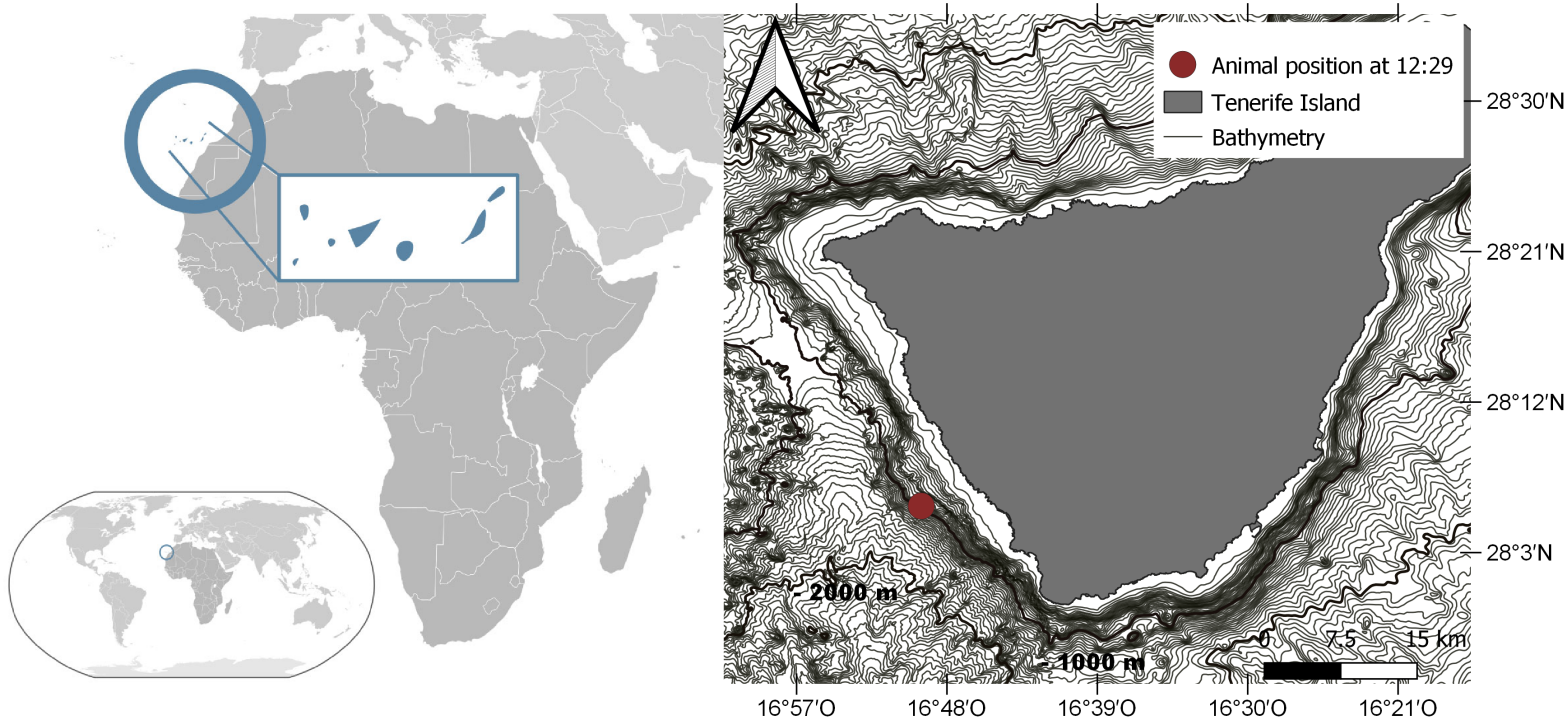
Special Area of Conservation (SAC) “Franja Marina de Teno‐Rasca” (European Habitats Directive 92/43/EEC), southwest off Tenerife, where the Asociación Tonina caught the injured pilot whale calf (28°05′79.13″ N, 16°49′53.63″ W).

**FIGURE 2:**
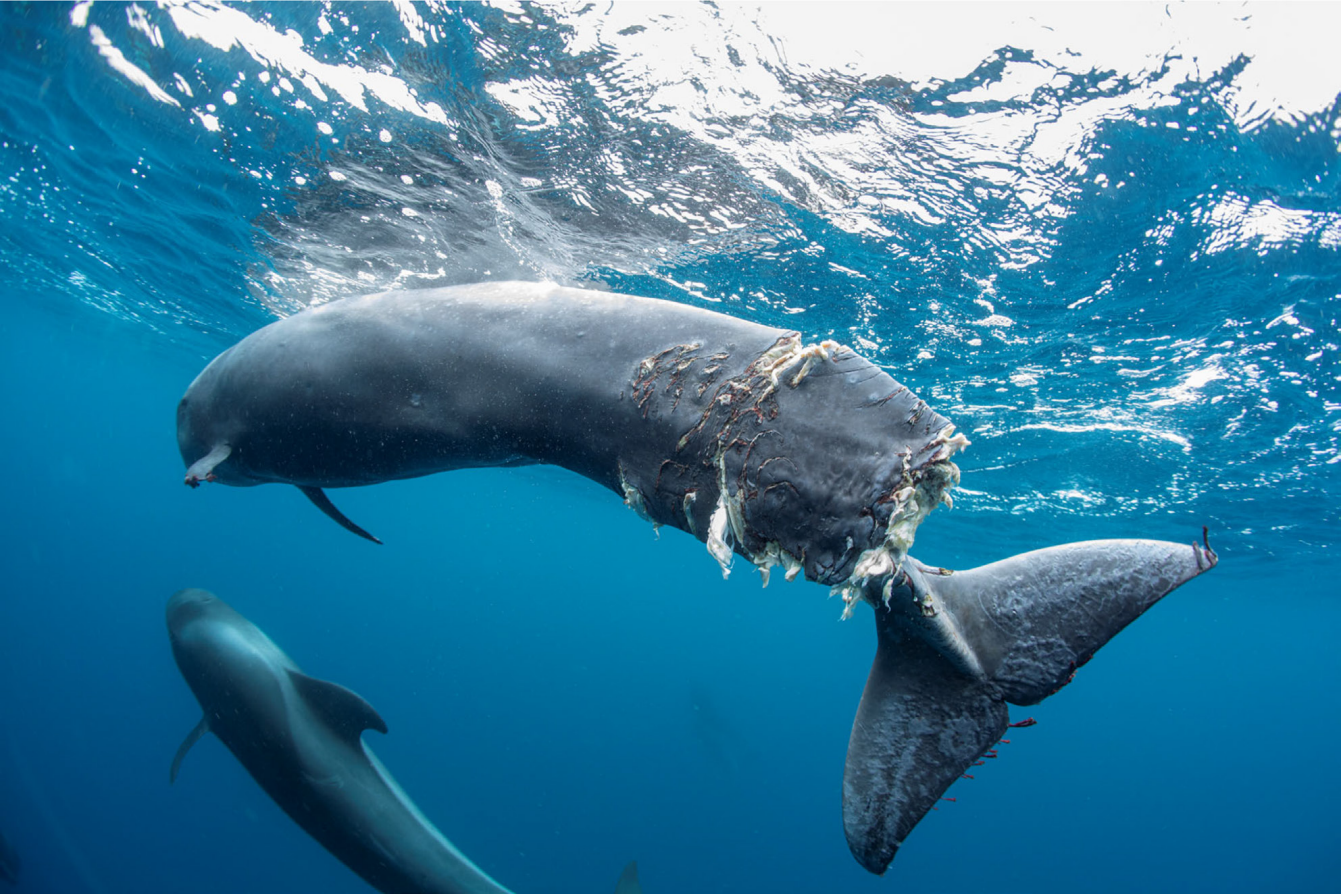
Injured young specimens of *Globicephala macrorhynchus* named “Hope” found in southwest coast off Tenerife, 24/03/2019. © Francis Pérez.

During a survey campaign conducted on 24th March 2019 in the Special Area of Conservation (SAC) “Franja Marina de Teno‐Rasca” (European Habitats Directive 92/43/EEC, Figure 1), southwest off Tenerife, the Asociación Tonina received a radio alert from a whale‐watching boat about an injured pilot whale calf that had been sighted in the area (28°05′79.13″ N, 16°49′53.63″ W). Permission for the operation and the handling of the animal was granted by the Spanish Ministry of Environment. The association's team and a professional underwater photographer with administrative permission to photograph cetaceans in the SAC navigated to the position. A pilot whale calf was located resting on surface (28°06′04.0″ N, 16°49′12.2″ W). The animal had difficulties to swim and to remain upright. Acoustic and photoidentification data, underwater photos and videos (Canon 5D MKIV, Seacam housing), showed that the animal's caudal fin was almost completely cut off near the caudal peduncle (Figure 2; Appendix S1: video link 1). Simultaneously, three adults pilot whales were approaching, presumably alerted by the calls that the calf was producing repeatedly. The team proceed by sending the audiovisual material to the authorities, notifying them the critical situation of the animal. Once they confirmed that the veterinarian team from the Wildlife Recovery Center “La Tahonilla” (Cabildo of Tenerife) were moving to the area, we proceed according to the stranding surveillance protocol. Whale‐watching vessels in the area were asked to keep their distance. Four hours later, the veterinarian staff arrived in a Zodiac and assessed the health status of the calf in situ. The severity of the injuries made it impossible for the animal to survive in the wild on its own, so the chief veterinarian decided to euthanize it.

The maneuver was performed with two zodiacs, skippered by the qualified staff of both institutions. Then, the animal was captured with the help of a net stretched underneath the animal between the two boats and euthanized (intraperitoneal injection of 100 cc/130 kg of Dolethal (pentobarbital) with xylazine, to facilitate the quick diffusion of the anesthetic into the bloodstream). Few minutes later the animal perished, and his body was transferred to the Wildlife Recovery Center.

### 2.2 Photoidentification

Dorsal fin photographs of pilot whales accompanying the calf were compared with the Tenerife Island species photo‐identification catalog (Pimentel et al., 2023). This catalog is regularly updated and contains photo cards of more than a thousand recognized individuals taken on field cruises from 2014 to date.

### 2.3 Sample collection and preparation

Necropsy was conducted by staff of the University Institute of Animal Health and Food Safety (IUSA), Veterinary School, University of Las Palmas de Gran Canaria (ULPGC), following standardized protocols (Kuiken & García‐Hartmann, 1991).

Samples of skin, blubber and muscle were collected from the surface body in front of the dorsal fin of the animal and were storaged at −80°C until analysis. Skin (N = 1) was removed and used to analyze stable isotopes, while blubber was separated into three layers: the outer layer closest to the skin (0.9 cm), the middle layer (0.4 cm), and the inner layer (1 cm) closest to the muscle. These samples were used for the FA determination. The stomach contents were temporarily frozen at −20°C until their analysis in the laboratory.

### 2.4 Stomach content identification

Cephalopod beaks were cleaned and preserved in 70% ethanol. They were used for specific identification according to Clarke's (1986) and Pérez‐Gándaras' (1983) methods. The lower beaks were identified using the available specific literature (Clarke, 1986; Pérez‐Gándaras, 1983; Roper, 1966; Xavier & Cherel, 2021), and two cephalopod beaks reference collection from the Institute de Ciencias del Mar (ICM‐CSIC, Barcelona), the general collection and the AFOC (Shape Analysis of Cephalopods' Beaks) collection, for corroboration of identifications. The Lower Rostral Length (LRL), Lower Crest Length (LC), Lower Hood Length (LHL), and Lower Width (LW) of the cephalopod lower beaks was measured (vernier calipers, 0.1 mm), and the following indexes were calculated: LC/LHL, LHL/LRL, LRL/LW, LH/LW, LC/LW, to compare them with the described in literature (Clarke, 1986; Pérez‐Gándaras, 1983). Allometric equations for dorsal mantle length (ML) and mass (W) estimation for cephalopods were taken from the literature (Clarke, 1986; Lu & Ickeringill, 2002; Pérez‐Gándaras, 1983; Rodhouse et al., 1990; Roper, 1966).

### 2.5 Stable isotopes analyses

Lipids were extracted from the skin sample using 4 mL of cyclohexane for 1 h; the sample was centrifuged for 5 min at 4000*g*, and the supernatant containing the lipids discarded (Méndez‐Fernández et al., 2012). Then, the sample was dried in an oven at 45°C, for 48 h, and 0.3 ± 0.05 mg subsamples of lipid‐free dried powder were finally weighed in tin capsules for stable isotope analyses. These analyses were performed with an isotope ratio mass spectrometer (IRMS‐MAT 253, Finnigan™) coupled to a continuous flow interface (ConFlo III) in the lab facilities of CACTI‐University of Vigo (Vigo, Spain). The results are presented in the usual δ notation relative to Vienna PeeDee Belemnite Standard (VPDB) for δ^13^C and atmospheric N_2_ for δ^15^N, in parts per thousand (‰) expressed as δX^y^ = [(Rsample − Rstandard)/(Rstandard)], where *X* is the element, *y* is the atomic mass of the stable isotope, and *R* is the ratio of heavy to light isotopes.

### 2.6 Fatty acid profile analyses and stratification index

Lipids were independently extracted from each blubber sample (inner, middle, and outer layer) with chloroform–methanol (2:1, v/v) following a modified version of Folch et al'. (1957) method as described by Galindo et al. (2022). An aliquot of 1 mg of lipid was subjected to acid‐catalyzed transmethylation (Christie, 1982), and the obtained fatty acid methyl esters (FAME) were purified by thin layer chromatography (Christie & Han, 2010). The resultant FAME were resuspended in hexane with butylated hydroxytoluene (BHT) and stored at −20°C until further analysis.

For the identification and quantification of FA, a TRACE‐GC Ultra gas chromatograph (Thermo Scientific, Milan, Italy) equipped with an on‐column injection, a flame ionization detector (FID) set at 250°C, and a Supelcowax™ 10 fused silica capillary column (30 m × 0.32 mm I.D. 0.25 μm thickness; Supelco Inc., Bellefonte, Pennsylvania, USA) was used. Helium was employed as the carrier gas, and temperature programming began at an initial temperature of 50°C, increased at 40°C/min to 150°C, from 150°C to 200°C at 2°C/min, to 214°C at 1°C/min, and to a final temperature of 230°C at 40°C/min, which was maintained for 5 min. A mixture of authentic standards (Mix C4‐C24 and PUFA No. 3 from menhaden oil [Sigma Aldrich, Darmstadt, Germany]) and a well‐characterized cod roe oil were used to identify individual FAME. If needed, the identity of FAME was confirmed by GC–MS (DSQ II, Thermo Scientific).

To identify the differences in FAME composition between layers, a stratification index (SI) was calculated, following Guerrero and Rogers (2017) and Olsen and Grahl‐Nielsen (2003). This index is based on the composition differences between the outer and inner layers of the blubber showing a possible stratification. SI is calculated taking the percentage concentration of each FA in the outer and inner layers, according to the equation by Olsen and Grahl‐Nielsen (2003): $\mathrm{SI}=\left(F_{o}-F_{i}\right)/\left(\left(F_{o}+F_{i}\right)/2\right)$, where *F*_o_ is the proportion of a FA in the outer layer, and *F*_i_ is the proportion of the same FA in the inner layer.

## 3 RESULTS

### 3.1 Necropsy results and photo‐identification

Preliminary necropsy results revealed that the euthanized animal was a 213 cm long, less than one‐year‐old female (Kasuya & Marsh, 1984). The causes of the injures remains under investigation. This animal was named ‘Hope’ (without code) and the accompanying adults were photoidentified as: ‘Alejandrina’ (GM_CC_I_443) and ‘Tere’ (GM_CC_D_712), both resident animals that have been repeatedly sighted forming part of well‐known groups in the south‐west of Tenerife (Pimentel et al., 2023). Hope's presumed mother (animal that stood next to her, traveling together) could not be identified, because her dorsal fin had no marks that helped in its identification.

### 3.2 Stomach contents analysis

A total of 144 cephalopod beaks remained in the stomach. It contained 77 upper and 67 lower beaks, a number of cephalopod eye lenses and sucker rings, all of them mixed with milk. 77.6% (52 individuals) of lower beaks were incomplete, with only the hood remaining, broken wings or broken crest, making it difficult to identify them correctly at species level. Nevertheless, a total of 15 different taxa of oegopsid squids were identified from the lower beaks. The list of cephalopod species recovered from the stomach is shown in Table 1.

**TABLE 1:** Cephalopod species identified from the beaks found in the stomach contents of a calf pilot whale euthanized on the island of Tenerife.

Family/species	*N*	% *N*	ML (mm)	W (gr)	Geog. Distrib.	Reference	Depth (m)
**Chiroteuthidae**							
*Asperoteuthis acanthoderma* (Lu, 1977)	1	1.51	56.36	184.39	Indo‐Pacific, Atl.	Clarke (1986) for *Mastigoteuthis* sp.	200–1100^a^
**Cranchiidae**							
Cranchid not id.	1	1.51	63.11	4.24		Clarke (1986) for Cranchiinae spp.	>2000
**Enoploteuthidae**							
*Enoploteuthis leptura* (Leach, 1817)	22	33.3	31.71–223.83	1.80–429.23	Tropical‐subtropical Atl.	Lu and Ickeringill (2002) for *Enoploteuthis* spp.	0–1620^b^
**Histioteuthidae**							
Histioteuthid not id.	1	1.51	40.81	39.02		Clarke (1986) for Histioteuthidae.	0–4000
*Histioteuthis bonnellii* (Férussac, 1835)	8	12.1	26.34–159.13	11.27–27.64	Atl., SW Pacific, S Indian, Mediterranean	Pedà et al. (2022)	150–4000
*Histioteuthis corona* (N. A. Voss & G. L. Voss, 1962)	2	3.03	23.27–57.47	15.87–72.3	Tropical‐subtropical Atl.	Clarke (1986) for Histioteuthidae.	100 to >1500
*Stigmatoteuthis arcturi* G. C. Robson, 1948	4	6.06	25.32–66.54	68.67–294.23	Tropical‐subtropical Atl.	Clarke (1986; for ML >180 mm)	200–1000
**Lepidoteuthidae**							
*Lepidoteuthis grimaldi* Joubin, 1895	6	9.09	30.87–86.49	120.30–203.86	Cosmopolitan tropical‐subtropical	Lu and Ickeringill (2002)	200–4000
**Bathyteuthidae**							
*Bathyteuthis abyssicola* Hoyle, 1885	1	1.51	32.63	3.09	Cosmopolitan	Clarke (1962)	700–2500
**Ommastrephidae**							
Ommastrephid not id.	5	7.57	0.94–6.60	187.96–1570.92		Clarke (1962)	0–2000
*Hyaloteuthis pelagica* (Bosc, 1802)	7	10.6	44.47–75.10	15.80–170.99	Subtropical Atl., Pacific	Clarke (1986)	15–800
*Ommastrephes caroli* (Furtado, 1887)	2	3.03	114.47–321.83	40.50–113.86	N Atl.	Agus et al. (2021)	0 to >1000^c^
*Sthenoteuthis pteropus* (Steenstrup, 1855)	2	3.03	48.99–98.24	15.92–184.30	Tropical‐subtropical Atl.	Guerra‐Marrero (2017)	0–1200
*Todarodes sagittatus* (Lamarck, 1798)	2	3.03	27.58–51.15	31.29–157.01	NE and S. Atl.	Clarke (1962)	0 to >1000. In North Africa, 350–700 m.
*Todaropsis eblanae* (Ball, 1841)	2	3.03	55.99–66.16	6.58–7.50	E Atl.	Pérez‐Gándaras (1983)	20–850
*N* TOTAL	66						

Abbreviations: Atl, Atlantic; Geog. Distrb., Geographic distribution of the species from Jereb and Roper (2010); ML, Estimated dorsal mantle length in mm; *N* %, percentages of beaks found for this species; *N* total, total number of beaks in the stomach; *N*, total number of beaks found for this species; W, Estimated weight in grams. References used for cephalopod ML regressions.

^a^ In Judkins et al. (2009); Joseph et al. (2015).

^b^ In Roper (1966).

^c^ In Young and Vecchione (2018).

The family with the highest species diversity in the stomach content was the Ommastrephidae Steenstrup, 1857 with six species (30.29% of the total), followed by Histioteuthidae Verril, 1881 with four species (22.72%). Other families including Chiroteuthidae Gray, 1849, Cranchiidae, Enoploteuthidae Pfeffer, 1900, Lepidoteuthidae Pfeffer, 1912, and Bathyteuthidae Pfeffer, 1900, were represented only by one species (Table 1). In addition, the three families best represented in number were the enoploteuthids (42.42%), followed by the ommastrephids (30.3%), and the histioteuthids (22.77%). *Enoploteuthis leptura* (Leach, 1817) (Cephalopoda: Enoploteuthidae) was the most abundant species in stomach contents (*N* = 22; 33.33% of the total), followed by *Histioteuthis bonnellii* (Férussac, 1834) (Cephalopoda: Histioteuthidae) (*N* = 8; 12.12%), and *Hyaloteuthis pelagica* (Bosc, 1802) (Cephalopoda: Ommastrephidae) (*N* = 7; 10.60%).

### 3.3 Trophic markers analyses

Stable isotopes from skin samples showed values of δ^15^N of 11.55‰ and −17.99‰ for δ^13^C. A total of 31 FA with values ≥0.1% were identified in the blubber samples (Table 2). Among them, five FA including oleic acid (18:1n‐9, 38.09 ± 3.08%), palmitic acid (16:0, 12.38 ± 1.58%), palmitoleic acid (16:1n‐7, 11.87 ± 0.88%), eicosenoic acid (20:1n‐9, 5.33 ± 0.21), and docosahexaenoic acid (22:6n‐3, 4.47 ± 1.22%), were the most abundant ones representing more than 70% of the total (Table 2). Overall, FA families were dominated by monounsaturated fatty acids (MUFA; 65.11 ± 3.57%); followed by saturated fatty acids (SFA; 20.23 ± 2.16%), and polyunsaturated fatty acids (PUFA; 13.17 ± 3.5%). Individually, all LC‐PUFA except 20:2n‐6, were notably more abundant in the inner layer than in the outer one. Thus, the SI values obtained were 20:3n‐3 (−0.21), 20:4n‐3 (−0.20), 20:5n‐3 (−0.40), 22:5n‐3 (−0.38), 22:6n‐3 (−0.55), and 20:4n‐6 (−0.04), 22:2n‐6 (−2), 22:4n‐6 (−0.24), and 22:5n‐6 (−0.26) (Table 2). Interestingly, most ≤C20 MUFA, including 14:1, 16:1, 17:1, and 18:1 were more abundant in the outer layer. As a consequence, total PUFA (SI = −0.29) and total SFA (SI = −0.15) were substantially more abundant in the inner layer, whereas total MUFA was in the outer layer (SI = 0.11).

**TABLE 2:** Fatty acid (FA) profiles of the inner, middle, and outer blubber layers (% of total FA) from a lactating *Globicephala macrorhynchus* from Tenerife.

Fatty acid	Inner	Middle	Outer	Mean ± SD	SI
14:0	3.64	3.28	3.29	3.41 ± 0.21	−0.10
14:1n‐5	0.55	0.68	0.59	0.61 ± 0.07	0.08
15:0	0.61	0.51	0.63	0.59 ± 0.06	0.04
16:0	14.17	11.17	11.81	12.38 ± 1.58	−0.18
16:1n‐9	1.13	1.13	1.42	1.23 ± 0.17	0.23
16:1n‐7	11.07	12.82	11.72	11.87 ± 0.88	0.06
16:2n‐4	0.77	0.89	0.91	0.86 ± 0.08	0.17
17:0	0.67	0.56	0.60	0.61 ± 0.05	−0.10
17:1n‐7	1.09	1.24	1.32	1.22 ± 0.12	0.20
18:0	3.55	2.95	3.23	3.24 ± 0.3	−0.09
18:1n‐9	34.79	38.59	40.89	38.09 ± 3.08	0.16
18:1n‐7	3.80	3.11	3.86	3.59 ± 0.42	0.02
18:1n‐5	0.41	0.40	0.37	0.39 ± 0.02	−0.11
18:2n‐6	0.99	1.11	1.11	1.07 ± 0.07	0.11
18:3n‐4	0.31	0.33	0.34	0.33 ± 0.02	0.10
18:3n‐3	0.33	0.38	0.34	0.35 ± 0.02	0.03
20:1n‐11	1.38	1.38	1.55	1.44 ± 0.1	0.12
20:1n‐9	5.57	5.23	5.19	5.33 ± 0.21	−0.07
20:1n‐7	0.35	0.30	0.29	0.31 ± 0.04	−0.20
20:2n‐6	nd	0.44	0.40	0.28 ± 0.24	2.00
20:4n‐6	1.15	1.29	1.11	1.18 ± 0.10	−0.04
20:3n‐3	0.40	0.35	0.32	0.36 ± 0.04	−0.21
20:4n‐3	0.35	0.29	0.29	0.31 ± 0.04	−0.20
20:5n‐3	1.87	1.81	1.25	1.64 ± 0.34	−0.40
22:1n‐11	0.70	0.57	0.63	0.64 ± 0.06	−0.10
22:1n‐9	0.34	0.27	0.31	0.31 ± 0.04	−0.10
22:2n‐6	0.46	nd	nd	0.15 ± 0.27	−2.00
22:4n‐6	0.34	0.31	0.27	0.31 ± 0.04	−0.24
22:5n‐6	0.46	0.31	0.36	0.38 ± 0.08	−0.26
22:5n‐3	1.70	1.57	1.16	1.48 ± 0.28	−0.38
22:6n‐3	5.63	4.60	3.20	4.47 ± 1.22	−0.55
UK	1.05	1.85	1.23		
ΣSFA	22.64	18.48	19.57	20.23 ± 2.16	
ΣMUFA	61.18	66.01	68.14	65.11 ± 3.57	
ΣPUFA	14.78	13.66	11.06	13.17 ± 1.90	
Σn‐6PUFA	3.41	3.46	3.24	3.37 ± 0.11	
Σn‐3PUFA	10.29	8.99	6.56	8.61 ± 1.89	
n‐3/n‐6	3.02	2.60	2.03	2.55 ± 0.49	
Σn‐3LC‐PUFA	9.96	8.61	6.22	8.26 ± 1.89	

Abbreviations: nd, not detected; SI, stratification index; UK, Unknown FA; ΣMUFA, total monounsaturated FA; Σɷ‐3 LC‐PUFA, total omega‐3 long‐chain PUFA; Σɷ‐3, total omega‐3 PUFA; Σɷ‐6, total omega‐6 PUFA; ΣPUFA, total polyunsaturated FA; ΣSFA, Total saturated FA.

## 4 DISCUSSION

### 4.1 Stomach content identification

A large number of lower beaks (77.6%) were badly damaged, making correct identification to species level extremely difficult. The results show that the calf had fed on a variety of 15 different species of oceanic squid, including enoploteuthids (13.3%) and several species of histioteuthids (19.4%). All species found in this study have been previously reported in the region, except the chiroteuthid *Asperoteuthis acanthoderma* (Lu, 1977) (Cephalopoda: Chiroteuthidae) (Cherel, 2021; Young & Roper, 2018) which until now had only been cited in the western Atlantic, in the Gulf of Mexico, and Florida. This result suggests the possibility of its presence on both sides of the subtropical Atlantic, extending its currently known distribution.

The diel vertical migrant species of the families Pyroteuthidae Pfeffer, 1912, Enoploteuthidae, and Onychoteuthidae Gray, 1847 represent the core of the cephalopod community in shallow (<200 m) nocturnal oceanic waters in the subtropical Atlantic (Ariza et al., 2016; Escánez et al., 2022). Although they are also abundant in deeper layers, non‐migratory phases or mesopelagic species are notable in this area (Escánez et al., 2022). This is in line with the high percentage of enoploteuthids found in the stomach, which suggests a shallow foraging habitat of this young pilot whale. *Enoploteuthis leptura*, the largest species of the family, performs vertical migrations from mesopelagic to epipelagic depths, where they concentrated in the first 20 m during the night (Jereb & Roper, 2010). The ML range for two of the *E. leptura* encompasses larger animals than those cited in the literature (maximum ML of 92 mm; Jereb & Roper, 2010; see Table 1). This can be due to the fact that there are no specific allometric equations for this particular species, having used equations pertaining to *Enoploteuthis* spp. (Lu & Ickeringill, 2002) in the present study. In addition, most beaks were quite eroded, preventing the measurement of the total length of their wings, which might distort the results of the calculated indices and, therefore, the correct identification of the specimens.

*Enoploteuthis leptura* is a foraging species for several predators such as fishes (e.g. *Thunnus obesus* (Lowe, 1839), Teleostei: Scombridae, *Thunnus alalunga* (Bonnaterre, 1788), Teleostei: Scombridae, *Ruvettus pretiosus* Cocco, 1833, Teleostei: Gempylidae), cetaceans (*Kogia* spp.), or seabirds (e.g. petrels) (Bello, 1999; Ravache et al., 2020; Staudinger et al., 2013; Vaske et al., 2012). Similarly, the majority of the histioteuthids (*H. bonnellii*, *H. corona*, *Stigmatoteuthis arcturi* G. C. Robson, 1948) (Cephalopoda: Histioteuthidae) were represented in the stomach content by small individuals (23–57 mm of ML). These sizes correspond to juvenile individuals of each species as ML maximum sizes of these species are between 50 and 330 mm. This young pilot whale fed mostly on small cephalopods (small species and juvenile of large species), probably due to the animal's small size and therefore its ability to catch prey more in line with its size, as well as its swimming and diving abilities. Histioteuthids are distributed throughout the water column showing ontogenetic migration to deeper waters during their growth (Judkins & Vecchione, 2020; Quetglas et al., 2010) and, predictability, the individuals registered here (23–57 mm of ML) were at shallowest depths.

Including our present results, a total of 22 cephalopod species and three genera belonging to 10 families of Teuthida have been identified so far in the stomach contents of four pilot whales from the Canary Islands. Among them, ommastrephids, histioteuthids, and cranchids contain the higher number of species (Table S1). The Canary Islands holds a rich cephalopod community with 85 species belonging to 31 families (Escánez et al., 2021), which means that pilot whales' prey on about 25.9% of the total known cephalopod species in the Canaries. Compared to other regions where stomach contents of this cetacean species have been analyzed, Canary Islands present the highest cephalopods richness with only four specimens, followed by 27 pilot whales stranded in North Carolina (USA) of which a total of 11 cephalopods species were reported (Mintzer et al., 2008), and seven species identified in four pilot whales from California (USA) (Sinclair, 1992) (Table S1). This suggests that Canary Islands pilot whales are mainly teuthophagous predators, largely preying on a wide variety of squid species present in the area. However, predation on other types of prey, such as fish, cannot be completely ruled out for this species. The present study verifies that the diet of this young pilot whale was mainly based on squid.

Isotopic values of δ^15^N (11.55‰) of the young pilot whale were higher than expected values derived from the small oceanic squids found in its stomach (enoploteuthids and histioteuthids), but slightly lower than that of adult pilot whales from the region (12.2 ± 0.5; Escánez, 2019) and from the Iberian Peninsula (11.9 ± 0.3‰, Monteiro et al., 2017). The δ^15^N values for small squids of the families Enoploteuthidae and Histioteuthidae, dominants of stomach contents, ranged generally between 5.7 and 10‰ in Atlantic temperate waters (Cherel et al., 2009). The isotopic values of the nursing young pilot whale analyzed here (δ^15^N 11.55‰; δ^13^C −17.99‰) differ from adult individuals from Tenerife (δ^15^N 12.2 ± 0.5‰ and δ^13^C −15.8 ± 0.02‰). The results suggested that the isotopic values of the still‐suckling pilot whale are depleted in both, δ^15^N (Δ^15^N = −0.65‰) and δ^13^C (Δ^13^C = −2.19‰), compared with the mean values of adult individuals. Depletion in the δ^13^C in suckling calves (relative to the mother's tissues) is a common issue in several marine mammals that feed on lipid‐rich milks, since lipids are also depleted in ^13^C during biochemical discrimination (Borrell et al., 2016; Cherel et al., 2015; DeNiro & Epstein, 1977). In this case, pilot whales' (*Globicephala* spp.) milk has around 15–31% of lipid (Lockyer, 2007). On the other hand, δ^15^N values of nursing young that only feed milk should be one trophic level higher than mothers (Cherel et al., 2015). Unfortunately, we lack the information about δ^15^N values from the mother in our study. In any case, the isotopic values of the nursing young individual are the result of the isotopic mixing signatures of breast milk, food intake, and biochemical discrimination factors. It is to be expected that the influence from the maternal milk isotopic signature decreases over time with respect to isotopic influence of the food intake (mainly squids). Oftedal (1997) estimated ages of first ingestions of major solids for pilot whales in 6–12 months, while milk has been found in the stomach up to three years old individuals. These data emplace our nursing young animal with an estimated age of 6–12 months in its very early first food intakes.

The FA profile of the external blubber of this animal did not differ greatly from FA profiles previously reported in adult individuals from the same area during 2015 and 2017, where the same dominant FA (18:1n‐9, 16:0, 16:1n‐7, 20:1n‐9, and 22:6n‐3) were present in similar proportions, except 22:6n‐3, which was three times higher in the calf (Table 2) (Gil, 2018; work in preparation). Totals of MUFA and SFA were also found in similar amounts, but total PUFA was slightly higher in this calf (Table 2), possibly related to the importance of these FA during neurological, immune, and growth development of young animals (Parzanini et al., 2018). The great proportion of MUFA, primarily 18:1n‐9, in this species is probably related to its endogenous biosynthesis through the introduction of a first unsaturation into stearic acid (18:0) by means of a Δ9 desaturase activity (Castro et al., 2016; Hooker et al., 2001). Similarly, other major FA such as 16:1n‐7 has been previously reported to be endogenously biosynthesized from 16:0 in other aquatic mammals (Kirsch et al., 2000). MUFA have a key function in thermic isolation due to their lower melting point and their capacity for improving the cellular membrane fluidity in cold environments, being more abundant in the external layers in adults and juveniles. Furthermore, MUFA is an adaptive response to the pressure, an important adaptation for deep diving cetaceans as pilot‐whales (Burgess et al., 2018; Parrish, 2013). In addition, they are used as energy storage in many cetaceans. Similarly, short and medium chain SFA can be endogenously biosynthesized, also presenting thermic isolation functions, serving as energy storage and, as previously stated, as substrate to produce MUFA (Guerrero & Rogers, 2017). Notable variations in the percentage of FA in the inner and outer layers indicate a selective FA deposition during the development and growth of this young animal. Thus, all n‐3 and n‐6 LC‐PUFA that are typically ingested through the diet, are more prevalent in the inner blubber layer suggesting that this layer is more metabolically active in terms of fat storage and lipid deposition from the diet. By contrast, biosynthesized ≤C20 MUFA such as 14:1n‐5, 16:1n‐7, 18:1n‐9 and 20:1n‐11, were more abundant in the outer blubber layer.

Little is known about the diet of this species and even less about the ontogenetic changes during its early life stages. Opportunities such as the one described in this work are extremely rare. Our present results allow deepening our knowledge on the life cycle and bioecology of deep‐diving cetaceans, especially during their first life stages. This information is a valuable contribution to conservation policies. However, future studies, based on trophic markers such as isotopes and FA of both pilot whales and their potential preys in the area, together with the analysis of the stomach contents of a larger number of specimens, will help to better understand the needs and use of food resources on which depend the populations present in the oceanic islands of this region.

## AUTHOR CONTRIBUTIONS

**Amanda Luna:** Conceptualization (equal); data curation (equal); formal analysis (equal); investigation (equal); methodology (equal); resources (equal); writing – original draft (equal); writing – review and editing (equal). **Alejandro Escánez:** Conceptualization (equal); investigation (equal); methodology (equal); writing – original draft (equal); writing – review and editing (equal). **Jacobo Marrero:** Methodology (equal); writing – original draft (equal); writing – review and editing (equal). **Eva Íñiguez:** Formal analysis (equal); writing – original draft (equal). **José A. Pérez:** Methodology (equal); resources (equal); supervision (equal). **Pilar Sánchez:** Data curation (equal); methodology (equal); resources (equal); supervision (equal); writing – review and editing (equal).

## CONFLICT OF INTEREST STATEMENT

The authors declare no conflicts of interest.

## Supporting information


Appendix S1.

Table S1.

## Data Availability

Data available on request from the authors.
