# Supplementary material for: Early prey intake of a short‐finned pilot whale (Globicephala macrorhynchus Gray, 1846, Cetacea: Delphinidae) in the Canary Islands
Source: Ecol Evol. 2024 Mar 10;14(3):e11139. doi: 10.1002/ece3.11139 (PMC10925522; doi:10.1002/ece3.11139)
Supplement: Supplementary file 1 — Appendix S1. [file ECE3-14-e11139-s002.zip › Appendix_1_-_VIDEO.docx]

**Appendix 1.** Video of the young pilot whale injured at the southwestern coast of Tenerife, 24/03/2019. Video: © Francis Pérez.

<https://asociaciontonina.com/wp-content/uploads/2019/04/CALDERON-SOLO-SUBMARINA-LOGOS.mp4?_=1>
