# Supplementary material for: Early prey intake of a short‐finned pilot whale (Globicephala macrorhynchus Gray, 1846, Cetacea: Delphinidae) in the Canary Islands
Source: Ecol Evol. 2024 Mar 10;14(3):e11139. doi: 10.1002/ece3.11139 (PMC10925522; doi:10.1002/ece3.11139)
Supplement: Supplementary file 2 — Table S1. [file ECE3-14-e11139-s001.docx]

**Table S1.** Cephalopod species present in the stomach contents of *G. macrorhynchus* from literature. 1: Seagars & Henderson (1985), 2: Sinclair (1992), 3: Hernández-García & Martín (1994), 4: Bustamante et al. (2003), 5: Mintzer et al. (2008), 6: Fernández et al. (2009), 7: This study. CAN: species found in short-finned pilot whales from Canary Islands.

| **Family** | **Species** | **Reference** | **CAN** |
| --- | --- | --- | --- |
|  |  |  |  |
| **Bathyteuthidae** | *Bathyteuthis abyssicola* Hoyle, 1885 | 7 | + |
| **Brachioteuthidae** | *Brachioteuthis riisei* (Steenstrup, 1882) | 5 |  |
| **Chiroteuthidae** | *Asperoteuthis acanthoderma* (Lu, 1977) | 7 | + |
|  | *Chiroteuthis* spp*.* d'Orbigny [in A. Férussac & d'Orbigny], 1841 | 3 | + |
| **Chtenopterygidae** | *Chtenopteryx* spp*.* Appellöf, 1890 | 3 | + |
| **Cranchiidae** | *Cranchia scabra* Leach, 1817 | 3 | + |
|  | *Megalocranchia oceanica* (Voss, 1960) | 3,6 | + |
|  | *Taonius pavo* (Lesueur, 1821) | 3,5 | + |
|  | *Liocranchia reinhardti* (Steenstrup, 1856) | 5 |  |
| **Cycloteuthidae** | *Cycloteuthis sirventi* Joubin, 1919 | 3 | + |
|  | *Discoteuthis* spp*.* Young & Roper, 1969 | 3 | + |
| **Enoploteuthidae** | *Abralia veranyi* (Rüppell, 1844) | 5 |  |
|  | *Abraliopsis morisii* (Vérany, 1839) | 5 |  |
|  | *Enoploteuthis leptura* (Leach, 1817) | 7 | + |
| **Histioteuthidae** | *Stigmatoteuthis arcturi* Robson, 1948 | 5,7 | + |
|  | *Histioteuthis bonelli* (Férussac, 1835) | 7 | + |
|  | *Histioteuthis corona* (N. A. Voss & G. L. Voss, 1962) | 7 | + |
|  | *Stigmatoteuthis dofleini*Pfeffer, 1912 | 1,2 |  |
|  | *Histioteuthis heteropsis* (S. S. Berry, 1913) | 2 |  |
|  | *Histioteuthis meleagroteuthis* (Chun, 1910) | 3 | + |
|  | *Histioteuthis reversa* (Verrill, 1880) | 5 |  |
|  | *Histioteuthis* spp*.* d'Orbigny, 1841 | 4 |  |
| **Lepidoteuthidae** | *Lepidoteuthis grimaldi* Joubin, 1895 | 3,5,7 | + |
|  | *Pholidoteuthis adami* Voss, 1956 | 3 | + |
| **Loliginidae** | *Doryteuthis opalescens* (S. S. Berry, 1911) | 1,2 |  |
|  | *Loligo* spp*.* Lamarck, 1798 | 5 |  |
| **Lycoteuthidae** | *Selenoteuthis scintillans* Voss, 1959 | 3 | + |
|  | *Lycoteuthis* spp*.* Pfeffer, 1900 | 4 |  |
| **Mastigoteuthidae** | *Mastigoteuthis* spp*.* Verrill, 1881 | 5 |  |
| **Octopoteuthidae** | *Octopoteuthis deletron* Young, 1972 | 2 |  |
| **Ommastrephidae** | *Hyaloteuthis pelagica* (Bosc, 1802) | 7 | + |
|  | *Ommastrephes caroli* (Furtado, 1887) | 7 | + |
|  | *Sthenoteuthis pteropus* (Steenstrup, 1855) | 7 | + |
|  | *Sthenoteuthis* spp*.* Verrill, 1880 | 4 |  |
|  | *Todarodes sagittatus* (Lamarck, 1798) | 3,7 | + |
|  | *Todaropsis eblanae* (Ball, 1841) | 7 | + |
| **Gonatidae** | *Gonatus* sp*.* Gray, 1849 | 2 |  |
| **Onychoteuthidae** | *Onychoteuthis borealijaponica*Okada, 1927 | 2 |  |
|  | *Onykia robusta (Verrill, 1876)* | 1,2 |  |
|  | *Onykia* spp*.* Lesueur, 1821 | 4 |  |
